# The effects of fermented milk products (kefir and yogurt) and probiotic on performance, carcass characteristics, blood parameters, and gut microbial population in broiler chickens

**DOI:** 10.5194/aab-62-361-2019

**Published:** 2019-06-28

**Authors:** Mohammad Ghasemi-Sadabadi, Yahya Ebrahimnezhad, Abdolahad Shaddel-Tili, Vahid Bannapour-Ghaffari, Hashem Kozehgari, Mirmojtaba Didehvar

**Affiliations:** 1 Department of Animal Science, Faculty of Animal Science and Veterinary Medicine, Shabestar Branch, Islamic Azad University, Shabestar, Iran; 2 Department of Pharmacology, Faculty of Specialized Veterinary Sciences, Science and Research Branch, Islamic Azad University, Tehran, Iran; 3 Department of Food Science and Technology, Faculty of Food Science and Technology, Payame Noor University of Tabriz, East Azerbaijan, Tabriz, Iran

## Abstract

This study was conducted to determine the effects of fermented milk products and probiotic on performance, carcass characteristics, blood parameters, and gut microbial population in broiler chickens. A total of 480 one-day-old Ross 308 broilers were allocated to 30 floor pens in a completely randomized design with six treatments, five replicates, and 16 chicks (eight males and eight females) in each replicate. On the first day, the male and female chicks were weighed and divided by the feather sexing method so that the average body weight of chicks was approximately equal in each pen. Treatments consisted of six groups (including control): group 1 had a basal diet and normal drinking water, group 2 had a basal diet and probiotics (PrimaLac^®^) in drinking water as recommended by the manufacturer, group 3 had a basal diet and 2 % yogurt in drinking water, group 4 had a basal diet and 4 % yogurt in drinking water, group 5 had a basal diet and 2 % kefir in drinking water, and group 6 had a basal diet and 4 % kefir in drinking water. Chemical and microbiological characteristics of kefir and yogurt were measured after each production. The results showed that 4 % kefir, yogurt, and probiotic at the recommend level in water improved body weight gain, feed intake, and feed conversion ratio compared with other groups (P<0.05). The results indicated that treatment had a significant effect on the carcass yield, intestinal length, thigh yield, and abdominal fat in male and female chickens (P<0.05). There were no effects on total bacteria population but the lactobacilli and coliform bacteria populations showed increasing and decreasing trends, respectively, with 4 % kefir, yogurt, and probiotic supplementation at 28 and 42 d (P<0.05). In addition, blood glucose and total protein increased when using a high levels of kefir, yogurt, and probiotic in water, while cholesterol and LDL (low-density lipoprotein) concentrations were lower in 4 % kefir, yogurt, and probiotic at the recommended level. Consequently, the results of this study showed that the use of 4 % kefir, yogurt, and probiotic at recommended level in water had beneficial effects on the growth performance, intestinal bacteria population, and blood biochemical parameters in male and female broiler chickens.

## 1 Introduction

For many years, antibiotics were used as growth promoters in the poultry industry but recently these compounds have been prohibited due to their frequent use in animal diets and adverse effects on human and animal health conditions. Therefore, in many countries the prescription of antibiotics for animal diets has been prohibited (Apata, 2009; Shaddel-Tili et al., 2017). Hence, replacement of probiotics in the diets may decrease by use of antibiotics in ruminant and poultry diets (Fuller, 1998). Probiotics are a live microbial feed supplement that benefits the host by the intestinal microbial balance. Also, the utilization of probiotics in animal nutrition can provide healthy foods for humans (Denli et al., 2003). Similarly, researchers have defined probiotics as an alternative to antibiotics in animal diets (Fuller, 1998). Probiotics are increasingly adopted as an alternative to antibiotic growth promoters in poultry diets (Denli et al., 2003). In recent years, a large amount of research has been conducted on the use of probiotics in broiler chickens, and the majority of researchers reported that the use of probiotics improved the performance of broiler chicks (Jin et al., 1998b; Zulkifli el al., 2000; Kabir et al., 2004; Mountzouris et al., 2007). The previous studies observed that the use of probiotics has beneficial effects on poultry performance, For example, Khaliq and Ebrahimnezhad (2016) concluded that the use of probiotic from 1 to 42 d in diet improved the performance of broiler chicks. Also, Arslan and Saatci (2004) demonstrated that the use of 1.5 g kg${}^{-1}$ probiotic in water or diet improved live weight, feed consumption, and feed conversion efficiency of Japanese quail.

Fermented milk products are one of the important sources for transferring beneficial bacteria strains to the human digestive system (Itsaranuwat et al., 2003). Therefore, milk products contain important bacteria such as lactobacilli and *Bifidobacterium*, which have been used in different parts of the world as a probiotic supplement (Maity et al., 2008). Fermented milk dairy products from a variety of animals are perhaps the most common fermented foods worldwide (Farnworth, 2005). Hence, yogurt and kefir are fermented milk products that have been used as probiotics in animal and human nutrition (Farnworth, 2005).

Yogurt, which is known by many different names in different countries, is a fermented product which is familiar to consumers (Farnworth, 2005). Moreover, yogurt is a dairy product that contains useful bacteria such as lactic acid bacteria, *Lactobacillus bulgaricus*, *Streptococcus thermophilus*, and *L. delbrueckii* (Pelczar et al., 1986). In addition, yogurt can be effectively used as a probiotic in broiler chickens nutrition (Metchnikoff, 1998; Boostani et al., 2013). Yogurt also contains $10^{8}$ CFU mL${}^{-1}$ (where CFU means colony forming units) *Streptococcus thermophilus*, *Lactobacillus delbrueckii* (Boostani et al., 2013), 3.7 % protein, 3.4 % fat, and 4.7 % lactose (Nergiz and Seckin, 1998). Therefore, yogurt can have a direct impact on the health of the digestive tract by preventing the growth of pathogenic bacteria and maintaining the balance of beneficial bacteria (Metchnikoff, 1998). Similarly, Khan et al. (2011) compared probiotics, yogurt, and antibiotics and they reported that the use of yogurt-like probiotic has a significant effect on the performance of broiler chickens.

Kefir is another fermented milk product that is considered probiotic in animal or human nutrition (Yaman et al., 2006). Also, kefir belongs to the probiotic group originated from the Caucasus Mountains in Russia and is very popular in Middle Eastern drinks. Kefir is different from other fermented milk products because it comes from kefir grains that include a specific and complex mixture of lactic acid and acetic acid bacteria, and lactose-fermenting and nonfermenting yeast, which live in a symbiotic association (Rosa et al., 2017). The properties of kefir are derived from lactic acid, carbon dioxide, ethyl alcohol, and aromatic compounds that are obtained during fermentation (Rosa et al., 2017). Kefir is obtained by fermentation and the activity of lactobacilli such as *Lactobacillus lactis*, *Lactobacillus helveticus*, *Lactobacillus casei*, *Streptococcus* (*S. cremoris*, *S. lactis*), and yeasts (Otles and Cagindi, 2003; Toghyani et al., 2015). Usual kefir (fermented milk provided from kefir grains) contains almost 7.2 % protein, 6.6 % lactic acid, and less than 10 % fat. Also, kefir usually contains $9^{\text{8–10}}$ (CFU mL${}^{-1}$) of lactic acid bacteria (Rosa et al., 2017) and $10^{\text{6–7}}$ (CFU mL${}^{-1}$) yeast (Prado et al., 2015). Similarly, previous research stated that kefir contained proteins, polysaccharides, ethyl alcohol, lactic acid, fat, minerals, and vitamins (Magalhaes et al., 2011). Also, some strains of *Lactobacillus* and yeasts in kefir grain were previously reported as probiotics such as *Lactobacillus delbrueckii* subsp. *bulgaricus, L. acidophilus, L. plantarum, L. brevis, L. fermentum, L. casei, L. helveticus, L. lactis* subsp. *lactis, Streptococcus thermophilus*, and *Saccharomyces cerevisiae* (Yaman et al., 2006). Cho et al. (2013) reported that the use of kefir improved the growth performance of the broiler chickens. Also, an experiment conducted by Cenesiz et al. (2008) reported that the use of kefir has a beneficial effect on blood parameters of broiler chickens. However, kefir is less known than yogurt and it may contain bioactive ingredients that give it great health benefits, which means that kefir can be an essential probiotic product (Farnworth and Mainville, 2003; Farnworth, 2005).

Therefore, it seems that fermented milk products such as kefir and yogurt could be considered more useful probiotics by farmers; hence, the aim of this study was to determine the comparison of commercial probiotics with fermented milk probiotics on the performance of broiler chickens.

## 2 Materials and methods

### 2.1 Animals, breeding, and nutrition

All the chicks were kept under similar management conditions according to the Ross 308 strain catalogue. Animal handling and experimental procedures were performed according to the Guide for the Care and Use of Laboratory Animals by the Islamic Azad University Ethics Committee. A total of 480 one-day-old Ross 308 broiler chicks were used in this study. On the first day, male and female chicks were separated by the feather sexing method. At the beginning of the experiment, the male and female chicks were weighed so that the average body weight of chicks was approximately equal in each cage. Then the male and female chicks were allocated to 30 floor pens in a completely randomized design with six treatments, five replicates, and 16 chicks in each pen (eight males and eight females) throughout the experimental period, which was 42 d. Broiler chickens were breeding within floor pens and the dimensions of each pen were 1m×2 m.

A basal diet, considered control, was formulated according to the National Research Council recommendations (NRC, 1994) for the starter, grower, and finisher periods. The experimental diets are shown in Table 1.

**Table 1:** Composition of the basal diet (ingredients and nutrients) given to broiler chickens for 6 weeks.

Ingredient (%)	Starter	Grower	Finisher
	(1–10 d)	(11–24 d)	(25–42 d)
Corn	56.64	57.05	61.16
Soybean meal (44 % CP)	36.74	35.12	31.20
Soybean oil	0.95	3.20	3.22
Dicalcium phosphate (DCP)	1.89	1.65	1.53
Oyster shell	1.35	1.12	1.08
Sodium bicarbonate	0.26	0.26	0.26
Common salt	0.23	0.23	0.23
Vitamin premix${}^{1}$	0.25	0.25	0.25
Mineral premix${}^{2}$	0.25	0.25	0.25
Dextro-levo (DL) methionine	0.42	0.26	0.25
L-Lysine monohydrochloride	0.38	0.11	0.12
L-Threonine	0.64	0.50	0.45
Analyzed chemical composition			
Metabolizable energy (kcal kg${}^{-1}$)	2850	3000	3050
Crude protein (%)	22.14	20.95	19.54
Calcium (%)	1.05	0.90	0.85
Available phosphorus (%)	0.50	0.45	0.42
Sodium (%)	0.18	0.18	0.18
Potassium (%)	0.90	0.87	0.81
Chlorine (%)	0.17	0.17	0.17
Methionine + Cysteine (%)	1.07	0.90	0.86
Lysine (%)	1.43	1.18	1.09
Threonine (%)	0.94	0.80	0.74
Tryptophan (%)	0.30	0.29	0.26

${}^{1}$ Vitamin mixture provided per kilogram of diet: 3.60 mg vitamin A (retinol), 0.124 mg vitamin D${}_{3}$ (cholecalciferol), 18.20 mg vitamin E (alpha-tocopherol), 3.7 mg vitamin K${}_{3}$ (menadione), 3 mg vitamin B${}_{1}$ (thiamine), 15 mg vitamin B${}_{12}$ (cobalamin), 55 mg vitamin B${}_{3}$ (niacin), and 1 mg vitamin B${}_{7}$ (biotin). ${}^{2}$ Mineral mixture provided per kilogram of diet: 50 mg Mn (manganese), 47 mg Zn (zinc oxide), 25 mg Fe (iron), 7.5 mg Cu (copper sulfate), 2.6 mg I (iodine), and 0.40 mg Se (selenium).

Therefore, there were six experiment groups (including control): group 1 had a basal diet and normal drinking water, group 2 had probiotics (PrimaLac) in drinking water as recommended by the manufacturer, group 3 had 2 % yogurt in drinking water, group 4 had 4 % yogurt in drinking water, group 5 had 2 % kefir in drinking water, and group 6 had 4 % kefir in drinking water. The duration of the experimental period was 42 d. The feed and water were available ad libitum to broiler chickens.

The commercial probiotic in this study was PrimaLac^®^ (StarLabs Inc., Clarksdale, MO, USA), which is a multistrain commercial preparation in powder form ($2\times 10^{7}$ CFU g${}^{-1}$) that consists of *Lactobacillus acidophilus, L. casei, Enterococcus faecium*, and *Bifidobacterium bifidum* in drinking water.

### 2.2 Kefir preparation

Kefir was prepared from fresh cow milk so that the milk was pasteurized at 80 ${}^{\circ }$C for 30 min. After that, the pasteurized milk was cooled down to 25 ${}^{\circ }$C and then the kefir grains were added to whole milk at a proportion of 1:50 (w/v) for fermentation at temperatures from 22 ${}^{\circ }$C. At the end of fermentation, the kefir grains were removed by filtration from the kefir product (Rosa et al., 2017; Yaman et al., 2006).

### 2.3 Yogurt preparation

Fresh cow's milk was purchased and heated to 90 ${}^{\circ }$C for 10 min, cooled to 45 ${}^{\circ }$C, and inoculated with 3 % starter culture of *Streptococcus salivarius* subsp. *thermophilus* and *Lactobacillus delbrueckii* subsp. *bulgaricus*. After fermentation, the yogurt was cooled to room temperature (21 ${}^{\circ }$C) and stored in a refrigerator at 4 ${}^{\circ }$C for 12 h (Nergiz and Seckin, 1998).

### 2.4 Measurement of chemical and microbiological characteristics of kefir and yogurt

In this study, yogurt and kefir were produced every 7 days. After each production, the yogurt and kefir were stored in a refrigerator at 4 ${}^{\circ }$C. Regarding previous studies, it seems that cold storage can maintain the chemical and microbiological characteristics of these products during the 7 days (Irigoyen et al., 2005; Alirezalu et al., 2016). In addition, chemical and microbiological characteristics of kefir and yogurt were analyzed each time after production of fermented milk product. For this purpose, fat, protein, pH contents, lactic acid bacteria, and yeast in yogurt and kefir were determined according to Marshall (2005). The average values of chemical and microbiological characteristics in yogurt and kefir are shown in Table 2.

**Table 2:** Analysis of the chemical and microbiological composition of kefir and yogurt.

Fermented milk products	Lactic acid bacteria	Yeast	pH	Fat${}^{2}$	Protein${}^{2}$
	(log CFU g${}^{-1}$)	(log CFU g${}^{-1}$)		(g)	(g)
Kefir${}^{1}$	8.92±0.19	5.13±0.22	4.25±0.20	3.43±0.23	3.52±0.19
Yogurt${}^{1}$	8.21±0.22	2.6±0.23	3.91±0.22	3.13±0.26	3.79±0.18

${}^{1}$ Results are the average of six analyses ±SD. ${}^{2}$ Values of fat and protein are for 100 g yogurt and kefir samples.

### 2.5 Performance traits

At the end of each period, feed intake (FI), body weight gain (BWG), and feed conversion ratio (FCR) were measured. European broiler index (EBI) for the whole period (1 to 42 d) of breeding was calculated using the following equation.

European broiler index (EBI) = [average daily gain (grams of chick per day, g d${}^{-1}$) × viability (%) / (feed conversion ratio, g g${}^{-1}$) ×10] ×100.

### 2.6 Carcass traits

Six chicks (three male and three female) from each replicate were sampled (close to average weight) for carcass evaluations at 42 d of age, slaughtered, dissected manually, and weighed. The live body weight, eviscerated carcass, breast, thing, wings, abdominal fat, liver, and intestine were excised and weighed individually. The carcass yields were calculated as a percentage of the preslaughter live body weights of broiler chickens. The weights of these internal organs were expressed as a percentage of live body weight. Also, the size of the small intestine was expressed in centimeters.

### 2.7 Gut microbial population

At 24 and 42 d, six chicks (three male and three female) from each replicate were randomly selected for the measurement of intestinal microbial population.

For this purpose, 1 g of the composite gut sample from each chicken was diluted with 9 mL of 0.9 % saline solution and mixed in a vortex. Viable counts of bacteria in the gut samples were then conducted by plating serial 10-fold dilutions (in 1 % peptone solution) into lactobacilli de Man, Rogosa, Sharpe agar plates, and MacConkey agar plates (to isolate the *Lactobacillus* and coliform colonies). The lactobacilli de Man, Rogosa, and Sharpe agar plates were then incubated for 48 h at 37 ${}^{\circ }$C under anaerobic conditions. The *Lactobacillus* and coliform colonies were conducted immediately after removal from the incubator as described by Kang et al. (2013) and Khaliq and Ebrahimnezhad (2016). Also, for the enumeration of anaerobic bacteria, Reinforced Clostridial Agar was used. The plates were incubated in anaerobic jars (Oxoid) with Anaerogen (Oxoid) at 37 ${}^{\circ }$C for 48 h (Proietti et al., 2008).

Bacterial colony forming units (CFU) in the Petri dishes were counted using a colony counter. The counts were expressed as log${}_{10}$ colony forming units per gram of digesta (log${}_{10}$ CFU g${}^{-1}$).

### 2.8 Blood biochemical parameters

At the end of experimental period, six chicks (three male and three female) from each replicate were selected and blood samples were collected from the wing vein. All the samples were properly labeled and stored for further studies at -20 ${}^{\circ }$C. The plasma biochemical parameters including glucose, triglyceride (TG), cholesterol, low-density lipoprotein (LDL), high-density lipoprotein (HDL), total protein (TP), and albumin were determined by a Technicon RA-1000 auto-analyzer (Technicon Instruments Corporation, Tarrytown, NY, USA) and using the kit package (Pars Azmoon Co Tehran, Iran). Also, the plasma globulin concentration was determined by the difference between total protein and albumin (Coles, 1986).

### 2.9 Hematological parameters 

Six chicks (three male and three female) from each replicate were selected on day 42 by collecting blood samples from a wing vein. Blood samples obtained from all birds were drained into tubes with anticoagulant (EDTA, ethylenediaminetetraacetic acid, 1 mg mL${}^{-1}$ blood). Red-blood-cell (RBC) and white-blood-cell (WBC) counts were determined by using a hemocytometer according to Natt and Herrick, 1952. Packed cell volume (PCV) was determined using hematocrit tubes. The cyanmethemoglobin method as described by Benjamin (1978) was used to estimate the hemoglobin (Hb).

**Table 3:** The effects of using kefir, yogurt, and probiotic on performance of broiler chickens.

Traits	Period	Treatment							
		Control	Probiotic	2 %	4 %	2 %	4 %	SEM${}^{*}$	P value
			PrimaLac	yogurt	yogurt	kefir	kefir		
Body weight gain (g)									
	1–10 d	177.91	182.15	175.29	184.05	174.86	180.09	5.65	0.8225
	11–24 d	564.22${}^{c}$	592.24${}^{a}$	564.75${}^{c}$	586.79${}^{\mathrm{ab}}$	574.00${}^{\mathrm{bc}}$	593.11${}^{a}$	3.95	0.0001
	25–42 d	1617.47${}^{c}$	1679.30${}^{\mathrm{ab}}$	1606.47${}^{c}$	1691.20${}^{a}$	1628.88${}^{\mathrm{bc}}$	1690.16${}^{a}$	12.93	0.0002
	1–42 d	2359.61${}^{b}$	2453.69${}^{a}$	2346.51${}^{b}$	2462.04${}^{a}$	2377.74${}^{b}$	2463.36${}^{a}$	14.55	0.0001
Feed intake (g)									
	1–10 d	234.80	241.97	239.59	241.46	234.91	243.00	5.53	0.8255
	11–24 d	806.37${}^{c}$	836.74${}^{\mathrm{ab}}$	825.83${}^{b}$	837.36${}^{\mathrm{ab}}$	826.25${}^{b}$	841.00${}^{a}$	2.86	0.0001
	25–42 d	3139.72${}^{b}$	3245.96${}^{\mathrm{ab}}$	3157.51${}^{b}$	3246.56${}^{\mathrm{ab}}$	3140.19${}^{b}$	3306.98${}^{a}$	32.24	0.0063
	1–42 d	4180.89${}^{b}$	4324.67${}^{\mathrm{ab}}$	4222.94${}^{b}$	4325.38${}^{\mathrm{ab}}$	4201.36${}^{b}$	4390.98${}^{a}$	34.14	0.0018
Feed conversion ratio									
	1–10 d	1.32	1.33	1.37	1.31	1.34	1.35	0.03	0.9297
	11–24 d	1.42${}^{\mathrm{ab}}$	1.41${}^{b}$	1.46${}^{a}$	1.42${}^{\mathrm{ab}}$	1.43${}^{\mathrm{ab}}$	1.41${}^{\mathrm{ab}}$	0.02	0.0401
	25–42 d	1.94	1.93	1.96	1.91	1.92	1.95	0.02	0.8006
	1–42 d	1.77	1.76	1.80	1.75	1.76	1.78	0.01	0.6144
European broiler index									
	1–42 d	283.30	302.66	297.04	311.80	299.79	300.65	12.52	0.7371

${}^{a,b,c}$ Means in rows with same superscript do not differ significantly (P<0.05). ${}^{*}$ SEM: standard error of means.

### 2.10 Statistical analysis

Significant differences between mean values were separated by the GLM (general linearized model) procedure of SAS software (2003) and significant differences between treatments were separated using a Tukey range test at P<0.05.

## 3 Results

### 3.1 Performance

The effects of kefir, yogurt, and probiotic on BWG, FI, FCR, and EBI of the full rearing period on broiler chickens are shown in Table 3.

**Table 4:** The effects of using kefir, yogurt, and probiotic on carcass characteristics (based on live body weight) in male and female broiler chickens at 42 d.

Traits	Treatments							
	Control	Probiotic	2 %	4 %	2 %	4 %	SEM${}^{*}$	P value
		PrimaLac	yogurt	yogurt	kefir	kefir		
Male carcass characteristics								
Carcass yield (%)	72.65${}^{b}$	75.60${}^{\mathrm{ab}}$	73.69${}^{\mathrm{ab}}$	75.99${}^{\mathrm{ab}}$	73.40${}^{\mathrm{ab}}$	76.44${}^{a}$	0.81	0.0170
Abdominal fat (%)	1.58	1.46	1.46	1.46	1.54	1.46	0.04	0.1230
Liver (%)	2.97	2.66	2.48	2.97	3.12	3.16	0.22	0.2148
Intestine length (cm)	174.91${}^{b}$	182.05${}^{\mathrm{ab}}$	178.17${}^{\mathrm{ab}}$	181.71${}^{\mathrm{ab}}$	177.91${}^{\mathrm{ab}}$	182.58${}^{a}$	1.62	0.0217
Intestine yield (%)	3.51	4.25	3.44	4.17	3.80	4.13	0.23	0.1027
Breast yield (%)	37.72	38.02	37.05	37.35	37.40	39.69	0.81	0.2828
Thigh yield (%)	23.04${}^{b}$	27.64${}^{a}$	23.42${}^{b}$	26.30${}^{\mathrm{ab}}$	24.06${}^{\mathrm{ab}}$	24.15${}^{\mathrm{ab}}$	1.07	0.0459
Wings yield (%)	7.63	7.72	8.12	8.24	7.68	7.78	0.27	0.5132
Female carcass characteristics								
Carcass yield (%)	71.68${}^{c}$	75.40${}^{\mathrm{ab}}$	72.47${}^{\mathrm{bc}}$	76.74${}^{a}$	72.59${}^{\mathrm{bc}}$	76.42${}^{a}$	0.74	0.0002
Abdominal fat (%)	1.60${}^{a}$	1.34${}^{b}$	1.38${}^{\mathrm{ab}}$	1.38${}^{\mathrm{ab}}$	1.41${}^{\mathrm{ab}}$	1.37${}^{\mathrm{ab}}$	0.06	0.0463
Liver (%)	2.52	2.74	2.72	2.60	2.80	2.80	0.20	0.8947
Intestine length (cm)	175.52${}^{c}$	180.03${}^{\mathrm{ab}}$	176.88${}^{\mathrm{bc}}$	181.83${}^{a}$	179.93${}^{\mathrm{ab}}$	179.92${}^{\mathrm{ab}}$	0.97	0.0023
Intestine yield (%)	3.67	3.93	3.34	3.86	3.58	3.94	0.17	0.1244
Breast yield (%)	35.54	37.31	36.89	39.65	37.03	39.28	1.62	0.4810
Thigh yield (%)	24.91	26.43	24.35	26.07	23.96	25.46	0.76	0.2065
Wings yield (%)	7.75	7.70	7.65	7.83	7.72	7.82	0.23	0.9914

${}^{a,b,c}$ Means in rows with same superscript do not differ significantly (P < 0.05). ${}^{*}$ SEM: standard error of means.

The results showed that the experimental groups were not significant for effects on BWG in broilers at 1–10 d. But broiler BWG did differ between the experimental treatments at 11–24, 25–42, and 1–42 d (P<0.05). The use of 4 % kefir and probiotic in water had higher BWG than other groups at 11–24 d. Also, at 25–42 d, the use of 4 % kefir and yogurt in water was significantly higher for BWG compared to 2 % yogurt and control groups. In addition to the total experimental period, the treatments containing high levels of kefir, yogurt, and probiotic showed significantly high BWG than the other groups (P<0.05).

In this study, broiler FI did not differ between the experimental treatments at 1–10 d but broiler FI in other rearing periods was significantly affected (P<0.05).

At 11–24, 25–42, and 1–42 d, broilers supplemented with 4 % kefir had significantly higher FI compared with the control and low supplement levels of kefir and yogurt, which were not different between them. The results showed that FCR levels were not significantly different between the groups at different periods except for the second period (11–24 d). According to results, the use of probiotic at recommended levels was significantly lower FCR than 2 % yogurt in water (P<0.05).

Also, there were no significant differences in EBI between treatments during starter, grower, and finisher periods.

### 3.2 Carcass characteristics

The effects of kefir, yogurt, and probiotic on carcass characteristics at 42 d on male and female broiler chickens are shown in Table 4.

The results of this study showed that the carcass yield, thigh yield, and intestine length in male broilers – also carcass yield, abdominal fat, and intestine length in female chickens – were significantly affected by treatments (P<0.05). The 4 % kefir supplemented groups in male broiler chickens presented significantly higher carcass yield and intestine length compared with the control group (P<0.05). Also, the use of probiotic at recommended levels in water resulted in higher thigh yield than control groups in male broiler chickens.

Based on the results obtained from female chickens, it was observed that the birds under high levels yogurt and kefir treatment had significantly higher carcass yield compared with the control group (P<0.05). The results showed that the 4 % supplemented yogurt in water increased intestine length compared to control groups in the female birds. Also, abdominal fat was significantly lower (P<0.05) in the probiotic treatments than the control group.

### 3.3 Gut microbial populations

The effects of kefir, yogurt, and probiotic on gut microbial populations at 24 and 42 d on male and female broiler chickens are shown in Table 5.

**Table 5:** The effects of using kefir, yogurt, and probiotic on gut microbial population in male and female broiler chickens at 25 and 42 d.

Traits	Treatments							
	Control	Probiotic	2 %	4 %	2 %	4 %	SEM${}^{*}$	P value
		PrimaLac	yogurt	yogurt	kefir	kefir		
Male gut microbial population at 25 d								
Coliform bacteria (log CFU g${}^{-1}$)	4.08${}^{\mathrm{ab}}$	3.44${}^{b}$	4.15${}^{a}$	3.76${}^{\mathrm{ab}}$	4.13${}^{a}$	3.79${}^{\mathrm{ab}}$	0.15	0.0176
*Lactobacillus* (log CFU g${}^{-1}$)	5.06${}^{c}$	6.02${}^{\mathrm{ab}}$	5.36${}^{\mathrm{cb}}$	5.87${}^{\mathrm{ab}}$	5.02${}^{c}$	6.03${}^{a}$	0.14	0.0001
Total bacteria (log CFU g${}^{-1}$)	6.66	5.77	6.29	5.97	6.09	5.46	0.30	0.1448
Female gut microbial population at 25 d								
Coliform bacteria (log CFU g${}^{-1}$)	4.19${}^{a}$	3.54${}^{\mathrm{ab}}$	4.12${}^{\mathrm{ab}}$	3.51${}^{b}$	3.97${}^{\mathrm{ab}}$	3.53${}^{\mathrm{ab}}$	0.15	0.0077
*Lactobacillus* (log CFU g${}^{-1}$)	5.17${}^{b}$	6.06${}^{a}$	5.61${}^{\mathrm{ab}}$	6.01${}^{a}$	5.82${}^{\mathrm{ab}}$	6.09${}^{a}$	0.16	0.0157
Total bacteria (log CFU g${}^{-1}$)	6.25	6.12	6.14	6.08	6.11	6.06	0.17	0.9823
Male gut microbial population at 42 d								
Coliform bacteria (log CFU g${}^{-1}$)	6.20${}^{\mathrm{ab}}$	5.59${}^{\mathrm{ab}}$	6.47${}^{a}$	5.75${}^{\mathrm{ab}}$	6.14${}^{\mathrm{ab}}$	5.08${}^{b}$	0.28	0.0313
*Lactobacillus* (log CFU g${}^{-1}$)	5.93${}^{b}$	7.05${}^{a}$	5.84${}^{b}$	6.58${}^{\mathrm{ab}}$	6.23${}^{ab}$	7.09${}^{a}$	0.22	0.0022
Total bacteria (log CFU g${}^{-1}$)	7.27	6.47	6.85	6.40	7.09	6.53	0.55	0.8296
Female gut microbial population at 42 d								
Coliform bacteria (log CFU g${}^{-1}$)	5.98${}^{a}$	5.08${}^{b}$	5.80${}^{a}$	5.23${}^{b}$	5.92${}^{a}$	5.17${}^{b}$	0.06	0.0001
*Lactobacillus* (log CFU g${}^{-1}$)	6.73${}^{b}$	7.09${}^{\mathrm{ab}}$	6.81${}^{\mathrm{ab}}$	7.11${}^{a}$	6.73${}^{b}$	7.12${}^{a}$	0.07	0.0025
Total bacteria (log CFU g${}^{-1}$)	7.07	6.51	6.98	6.01	6.96	6.77	0.37	0.3679

${}^{a,b,c}$ Means in rows with same superscript do not differ significantly (P<0.05). ${}^{*}$ SEM: standard error of means.

These results indicate that treatments had an effect on lactobacilli and coliform bacteria populations in male and female broiler chickens at 25 and 42 d (P<0.05). In male broiler chickens, the coliform bacteria population decreased in the group treated with probiotic compared with broilers supplemented with 2 % yogurt and kefir in water. Also, the use of 4 % yogurt in water decreased coliform bacteria population in female broiler chickens at 25 d.

A significant increase in lactobacilli population was observed in the male broiler chickens that received high levels of kefir, yogurt, and probiotic at recommended levels compared with the control group (P<0.05).

Additionally, the results of this study showed that the supplement of 4 % kefir in female broiler chickens significantly increased lactobacilli population at 25 d (P<0.05). At 42 d the results indicated that supplemented 4 % kefir in water in male broiler chickens and 4 % kefir and yogurt and probiotic at recommended levels in female broiler chickens caused a decrease in coliform bacteria population. The supplement of 4 % kefir and probiotic at recommended levels in water significantly increased lactobacilli population at 42 d in male broiler chickens (P<0.05). Also, female broilers that received higher kefir and yogurt levels had higher lactobacilli population compared with 2 % kefir and control groups that were not different between them.

### 3.4 Blood biochemical parameter

The effects of kefir, yogurt, and probiotic in water on blood biochemical parameters at 42 d on male and female broiler chickens are shown in Table 6.

**Table 6:** The effects of using kefir, yogurt, and probiotic on blood biochemical parameters in male and female broiler chickens at 42 d.

Traits	Treatments							
	Control	Probiotic	2 %	4 %	2 %	4 %	SEM${}^{*}$	P value
		PrimaLac	yogurt	yogurt	kefir	kefir		
Male blood biochemical parameters at 42 d								
Glucose (mg dL${}^{-1}$)	205.75${}^{b}$	226.50${}^{a}$	209.25${}^{\mathrm{ab}}$	228.75${}^{a}$	223.25${}^{\mathrm{ab}}$	221.00${}^{\mathrm{ab}}$	4.35	0.0070
Cholesterol (mg dL${}^{-1}$)	120.00${}^{a}$	107.25${}^{\mathrm{ab}}$	117.00${}^{\mathrm{ab}}$	105.25${}^{b}$	107.75${}^{\mathrm{ab}}$	104.25${}^{b}$	3.05	0.0068
Triglyceride (mg dL${}^{-1}$)	25.50	21.25	23.25	24.25	25.25	23.25	1.47	0.3801
LDL${}^{2}$ (mg dL${}^{-1}$)	35.61	33.25	35.73	32.62	31.75	30.24	1.06	0.3360
HDL${}^{3}$ (mg dL${}^{-1}$)	75.43	69.25	74.50	71.14	73.00	70.52	3.16	0.7419
Total protein (g dL${}^{-1}$)	5.06${}^{\mathrm{ab}}$	5.55${}^{\mathrm{ab}}$	4.95${}^{b}$	5.68${}^{\mathrm{ab}}$	5.13${}^{\mathrm{ab}}$	6.37${}^{a}$	0.31	0.0412
Albumin (g dL${}^{-1}$)	2.70	3.29	3.13	3.18	3.13	3.23	0.22	0.4884
Globulin (g dL${}^{-1}$)	2.36	2.26	1.82	2.50	2.00	3.14	0.33	0.1430
Female blood biochemical parameters at 42 d								
Glucose (mg dL${}^{-1}$)	209.50${}^{b}$	221.25${}^{\mathrm{ab}}$	218.75${}^{\mathrm{ab}}$	221.75${}^{a}$	219.25${}^{\mathrm{ab}}$	222.25${}^{a}$	2.65	0.0317
Cholesterol (mg dL${}^{-1}$)	123.75${}^{a}$	114.75${}^{\mathrm{ab}}$	123.50${}^{a}$	112.75${}^{b}$	117.25${}^{\mathrm{ab}}$	112.75${}^{b}$	2.01	0.0015
Triglyceride (mg dL${}^{-1}$)	26.00	23.50	26.75	20.50	26.25	23.50	2.12	0.3276
LDL (mg dL${}^{-1}$)	37.00${}^{a}$	31.50${}^{b}$	36.50${}^{a}$	34.50${}^{\mathrm{ab}}$	34.25${}^{\mathrm{ab}}$	33.75${}^{\mathrm{ab}}$	1.05	0.0208
HDL (mg dL${}^{-1}$)	79.12	78.25	79.49	75.77	79.45	75.23	1.35	0.1358
Total protein (g dL${}^{-1}$)	5.08	5.53	4.99	5.93	5.15	5.92	0.32	0.3314
Albumin (g dL${}^{-1}$)	2.97	2.84	3.27	3.39	3.27	3.50	0.21	0.4735
Globulin (g dL${}^{-1}$)	2.11	2.69	1.72	2.54	1.88	2.45	0.41	0.5071

${}^{a,b,c}$ Means in rows with same superscript do not differ significantly (P<0.05). ${}^{1}$ SEM: standard error of means. ${}^{2}$ LDL: low-density lipoprotein. ${}^{3}$ HDL: high-density lipoprotein.

Blood glucose, cholesterol, and total protein concentrations in male broiler chickens were affected by treatments. Additionally, the treatments had an effect on blood glucose, cholesterol, and LDL concentrations in female broiler chickens (P<0.05). These results indicate that the supplementation with 4 % yogurt in male broiler chickens had higher blood glucose concentration compared with the control group. Also, the use of high levels of kefir and yogurt in water decreased blood cholesterol concentration than the control group in male broilers. The results of total protein showed a significant difference between treatment and control groups (P<0.05). The total protein concentration was significantly increased by the use of high levels of kefir in water compared with the control group in the male broilers (P<0.05).

In this experiment, the blood glucose concentration significantly increased with the addition of 4 % kefir and yogurt in the water (P<0.05). Also, the use of higher levels of kefir, yogurt, and probiotic at recommended levels in water resulted in significantly decreased blood cholesterol concentrations in female broiler chickens (P<0.05). Additionally, the utilization of probiotic at recommended levels in water resulted in significantly decreased blood LDL concentrations in female broiler chickens (P<0.05).

### 3.5 Blood hematological parameter

The effects of different levels of kefir, yogurt, and probiotic in the water on blood hematological parameters at 42 d on male and female broiler chickens are shown in Table 7.

**Table 7:** The effects of using kefir, yogurt, and probiotic on hematological parameters in male and female broiler chickens at 42 d.

Traits	Treatments							
	Control	Probiotic	2 %	4 %	2 %	4 %	SEM${}^{*}$	P value
		PrimaLac	yogurt	yogurt	kefir	kefir		
Male hematological parameters at 42 d								
White blood cell ($\times 10^{3}$ µL${}^{-1}$)	22.05	21.97	20.97	21.90	20.75	21.90	0.85	0.8106
Red blood cell ($\times 10^{6}$ µL${}^{-1}$)	3.08	2.98	2.75	2.78	2.75	2.71	0.42	0.9842
Hemoglobin (g dL${}^{-1}$)	8.12	9.47	8.27	9.00	8.37	8.95	0.46	0.3206
Packed cell volume (%)	29.50	29.75	28.25	29.25	29.75	29.25	1.26	0.9605
Female hematological parameters at 42 d								
White blood cell ($\times 10^{3}$ µL${}^{-1}$)	21.65	21.95	21.37	22.00	20.87	21.30	0.45	0.5302
Red blood cell ($\times 10^{6}$ µL${}^{-1}$)	2.40	2.07	2.29	2.31	2.21	2.49	0.27	0.8859
Hemoglobin (g dL${}^{-1}$)	7.90	7.55	8.20	7.32	7.70	8.22	0.61	0.8754
Packed cell volume (%)	28.25	27.75	26.75	28.00	26.00	26.50	0.94	0.4727

${}^{a,b,c}$ Means in rows with same superscript do not differ significantly (P<0.05). ${}^{*}$ SEM, standard error of means.

By examining the results, it can be concluded that the use of different levels of kefir, yogurt, and probiotic in water did not significantly affect WBC counts, total RBC count, PCV, and Hb at the end of the experiment.

## 4 Discussion

By examining the results obtained, it can be concluded that the use of 4 % kefir improved the BWG and FI of broiler chicks. In a similar study, Toghyani et al. (2015) reported that the use of 2 % kefir increased BWG and FI in broiler chickens. Also, in another experiment, Cenesiz et al. (2008) indicated that supplemented 7.5 % kefir increased BWG in broiler chickens. In general, the results of this study showed that using high levels of kefir in water can improve performance of broiler chickens; therefore, it can be stated that kefir is a natural product that contains some compounds like lactic acid bacteria and yeast (Farnworth, 2005). Therefore this product has been reported to have probiotic activity and can improve the performance of broiler chickens (Toghyani et al., 2015). Cho et al. (2013) demonstrated that broilers supplemented with 0.1 % fermented milk kefir in diets had significantly higher body weight at the end of the experiment compared to control groups. Kefir is a natural product containing a complex mixture of lactic acid bacteria and yeasts, which have been reported to be probiotics (Fuller, 1989; Toghyaniet al., 2015). Also, based on our experiments, we found that the use of 4 % yogurt in water improved BWG compared to 2 % kefir, yogurt, and control group in broiler chickens. Similarity, Boostani et al. (2013) and Khan et al. (2011) stated that the use of yogurt in water improved the BWG, FI, and FCR of broiler chickens. Sultan et al. (2006) reported that the use of 5 mL${}^{-1}$ yogurt in water increased BWG compared to control group and low yogurt level. Boostani et al. (2013) indicated that a higher *Lactobacillus* population in yogurt increase BWG and FI in broiler chickens. Additionally, this researcher states that the high BWG of broilers fed yogurt was related to the lactobacilli population in yogurt. Also, by improving intestinal microbial balance, probiotics beneficially influence growth performance. Significant improvement in the BWG and FCR obtained in this study supports the results of others (Lan et al., 2003; Panda et al., 2006) that probiotics improve broiler performance. Probiotics can also improve broiler performance by increasing the villous height in the small intestine (Gunal et al., 2006; Panda et al., 2006). Talebi et al. (2008) demonstrated that the use of probiotic (PrimaLac) in diets increased BWG in broiler chickens. Generally, according to the results, it seems that the use of 4 % kefir, yogurt, and probiotic at recommended levels can increase beneficial bacteria as well as decrease pathogenic bacteria in the intestine, which can enhance intestine health and also improve digestion and absorption. In a similar case, researchers have stated that probiotics can improve gut-beneficial microorganisms by inhibiting the growth of pathogens, and consequently cause better digestion and absorption of nutrients (Jin et al., 2000; Toghyani et al., 2015). In this research, the high BWG and FI have been related to some benefits derived from enzymes of lactobacilli and high digestion of food. Furthermore, probiotic compounds increase the metabolizable energy by increasing the digestibility of carbohydrates due to increasing the activity of the amylase enzyme. In addition, probiotics can cause a reduction in the activity of harmful enzymes such as urease and glycosidase (beta-glucuronidase and beta-glucosidase), which disturb the digestive tract of the bird (Jin et al., 2000).

Regarding the data, it can be concluded that the use of 4 % kefir in water improved carcass yield in male and female broiler chickens at the end of the experiment. Other related studies included projects by Toghyani et al. (2015), who reported that the use of 2 % kefir in the water had no significant effect on carcass yield in broiler chickens. It has been previously reported that kefir grains contained some strains of the lactobacilli and yeasts and have already been described to be probiotics. Also they are used in probiotic preparations (Fuller, 1989). Therefore, supplementing of kefir including *Lactobacillus* bacteria into broiler water or diet caused an increase in the live body weight (Cho et al., 2013). The results of this research were in agreement with findings of other authors (Sultan et al., 2006), who reported that the use of probiotic products improved the carcass characteristics of broiler chickens. Moreover, Khaliq and Ebrahimnezhad (2016) concluded that the use of probiotic at total experimental period increased carcass yield of broiler chickens. It seems that the higher carcass yield in kefir treatment in this study is related to the high BWG at end of the experimental period. Also, the researchers stated that the high carcass yield was probably due to the probiotic effects on the secretion of compounds such as organic acids that improved nutrient metabolism and increased carcass yields (Ahangari et al., 2013). It was observed that the supplementation of 4 % kefir and 4 % yogurt in water increased the length of the intestine in male and female broiler chickens. Therefore, it seems that the higher intestine length in this study probably related to receiving probiotic compounds like lactobacilli and yeast that improves digestion and absorption processes in the intestine of the broiler chickens. In a similar case, Tsirtsikos et al. (2012) reported that the use of probiotic in diet improved intestinal conditions. In contrast with our results, Toghyani et al. (2015) reported that the use of kefir in diet decreased the length of intestine in broiler chickens. In addition, this study results showed that the use of probiotic at recommended levels in water decreased abdominal fat in female broiler chickens. Similarly, Yusrizal and Chen (2003) and Paryad and Mahmoudi (2008) reported that supplementation of probiotic had produced a low level of abdominal fat in broiler chickens. Hence, it seems that the use of probiotic increased digestion and absorption due to higher intestinal microbial population and gut health, and the balance of absorbed nutrients has increased and caused decreasing abdominal fat. Concluding the same result, other researchers reported that the use of probiotic decreased abdominal fat in broiler chickens (Khan et al., 2011; Boostani et al., 2013). In addition, low abdominal fat in this study can be related to low acetyl-CoA carboxylase, which is one of the important enzymes to fatty acid synthesis (Santoso et al., 1995).

The results of this study indicate that the use of 4 % kefir in water increased lactobacilli population in male and female broiler chickens at 25 and 42 d. Also, the use of 4 % kefir in water decreased coliform bacteria population in male and female broiler chickens at 42 d. In the same case, Yaman et al. (2006) demonstrated that the use of kefir increased lactobacilli population and decreased coliform bacteria population in intestine microflora. On the other hand, the results of this experiment showed that the use of 4 % yogurt increased lactobacilli and decreased coliform bacteria in female broiler chickens at 25 and 42 d. Similarity, Boostani et al. (2013) state that the supplementation of yogurt in water decreased *Escherichia coli* and *Clostridium perfringens* bacteria in broiler chickens. Based on our experiments, we found that the probiotic supplementation at recommended levels increased lactobacilli population in female chickens at 25 d and male broiler chickens at 42 d. While the use of probiotic decreased coliform bacteria in male chickens at 25 d and female chickens at 42 d. The results of this research were in agreement with findings of other authors (Khaliq and Ebrahimnezhad, 2016) showing that the use of lactobacilli sources as probiotic increased lactobacilli population and decreased coliform bacteria population in the intestine of broiler chickens. Generally, it seems that kefir, yogurt, and probiotic, having a beneficial microbial population such as lactobacilli, reduce the intestinal pH in the bird, and the intestinal environment becomes inappropriate for the proliferation of pathogens and therefore reduces the harmful microbial population in the intestine. Also, the researchers reported that reduction of coliform bacteria population in the intestine can be attributed to the production of lactic acid and acetic acid and other compounds from microorganisms imported through probiotic supplements into the intestine, which inhibit pathogenic bacterial growth and helps beneficial bacteria to adhere and rapidly colonize the intestinal mucosa of the animal (Fuller, 1989). In addition, it should be noted that the presence of yeast in kefir and yogurt has reduced the population of coliform bacteria in the intestine. Similarly, Hassanein and Soliman (2010) showed that the use of *Saccharomyces cerevisiae* decreased coliform bacteria population in broiler chickens. Lin et al. (2011) reported that supplementation of probiotics decreased *E. coli* population in controlled groups of ducks. Other similar studies included projects by Khaliq and Ebrahimnezhad (2016) and Yaman et al. (2006), who showed that the supplementation of probiotics increased lactobacilli population in broiler chickens. Furthermore, *Lactobacillus* bacteria may play an important role in preventing the growth of harmful bacteria in the digestive tract of birds (Fuller, 1989). It seems that the use of probiotics and fermented milk products can increase the *Lactobacillus* bacteria population in the intestine of broiler chickens (Yaman et al., 2006; Khaliq and Ebrahinezhad, 2016; Jin et al., 1998a).

In the present study, the usage of high levels of kefir has been shown to increase blood glucose concentration in female broiler chickens. Also, the supplementation of 4 % yogurt in water increased blood glucose concentration in male and female broiler chickens. Moreover, the probiotic treatment showed higher blood glucose concentration in male broiler chickens. For this purpose, the researchers showed that using probiotic supplements can increase blood glucose levels. In the same results, Ghasemi-Sadabadi et al. (2016) reported that the use of probiotics from 1 to 42 d increased blood glucose concentration in broiler chicks. In another experiment, however, Pourakbari et al. (2016) observed that the probiotic had no significant effect on blood glucose concentration. Increasing blood glucose concentration in this study may be related to the increased absorption of nutrients from the intestinal mucosa due to histomorphological changes in the intestine (Awad et al., 2009). Also, the high glucose level can be related to digestion of nutrients due to intestine enzymes (Mountzouris et al., 2007; Jin et al., 2000). The results of this study showed that use of 4 % kefir in water decreased blood cholesterol concentration in male and female broiler chickens. Toghyani et al. (2015) reported that the use of kefir in water decreased blood total cholesterol in broiler chickens. Furthermore, Cenesiz et al. (2008) demonstrated that milk kefir had a significantly lower total lipid and cholesterol in broiler chickens. Furthermore, some scientists (Brashears et al., 1998) suggested that reduced blood cholesterol concentration induced by kefir could be attributed to the deconjugation of bile acids by *Lactobacillus* spp. Also, in this experiment low blood cholesterol concentration was observed by treating with 4 % yogurt in water. Similarity, Khan et al. (2011) indicated that the use of yogurt in water decreased blood cholesterol concentration in broiler chickens. Haque et al. (2017) observed that the use of yogurt in water had significantly lower blood cholesterol concentration than commercial probiotic and control group. Overall, our results are in agreement with other researchers (Ghasemi-Sadabadi et al., 2016; Pourakbari et al., 2016; El-Rahman et al., 2012; Kalavathy et al., 2003). These researchers reported that the supplementation of probiotic compounds decreased blood cholesterol in broiler chickens. Also, it is hypothesized that some bacterial probiotic strains are able to incorporate cholesterol into the bacterial cells, hydrolyze bile salts, or inhibit hydroxymethylglutaryl-CoA, the rate-limiting enzyme of cholesterogenesis, thus reducing cholesterol in the body pool (Kalavathy et al., 2003). Similarly, results from other research state that *Lactobacillus* and bifidobacteria could contribute to the regulation of blood cholesterol concentrations by the conjunction of bile acids. Since the excretion of deconjugated bile acids was enhanced and cholesterol plays a role as its precursor, more molecules are spent for recovery of bile acids (Ghasemi-Sadabadi et al., 2016; Pereira and Gibson, 2002). Also, regarding the data, it can be concluded that supplementation of probiotic in water reduced blood LDL concentration in female broiler chickens. Kalavathy et al. (2003) and Pourakbari et al. (2016) demonstrated that the use of probiotic decreased the blood LDL concentration in broiler chickens. Fukushima and Nakano (1995) observed an improvement in the serum HDL and decline in blood LDL and cholesterol with probiotic. The blood LDL concentration decrease might be due to anorexia or defective lipid metabolism (Ghasemi-Sadabadi et al., 2010). Also, 60 % to 70 % change in plasma cholesterol directly affected blood LDL (Fischbach, 2004). The hypercholesterolemia effects of probiotic can be attributed to the compounds of *Aspergillus oryzae* that are known to inhibit biosynthesis of cholesterol (Hajjaj et al., 2005). Also, the hypercholesterolemia effect of *Aspergillus oryzae* can be related to the association with a key enzyme as hydroxy-3-methylglutaryl coenzyme A-3 in the synthesis of cholesterol in chickens (Lee et al., 2006). In the present study, the results showed that the use of 4 % kefir in water increased blood total protein concentration compared to 2 % yogurt in male chickens. As previous research stated that kefir as a fermented milk product has a probiotic role, research indicates that kefir products contain a complex mixture of beneficial bacteria; therefore, it includes advantageous effects like a probiotic (Cho et al., 2013). Hence, high blood total protein concentration in 4 % kefir in water may be related to the high *Saccharomyces cerevisiae* in kefir compared to yogurt. The same studies (Paryad and Mahmoudi, 2008) showed that the use of higher *Saccharomyces cerevisiae *increased blood total protein concentration in broiler chickens. Also, El-Rahman et al. (2012) stated that supplementation of probiotic increased blood total protein concentration. Arslan and Saatci (2004) showed that the use of probiotic in water improved blood protein concentration in quail. In contrast to this study, researchers found that blood total protein concentration was not affected in broilers that were given probiotic (Toghyani et al., 2015).

The results of this research showed that the use of different levels of kefir, yogurt, and probiotic in water did not significantly affect hematological parameters at the end of the experiment. The results of this research were in agreement with findings of other authors (Cho et al., 2013; Al-Saad et al., 2014; Khan et al., 2011) showing that WBC counts, RBC counts, PCV, and Hb were not affected by supplementation of probiotic products, This inconsistency among research reports may be related to differences in probiotic types or number of bacterial species present in probiotic products (Al-Saad et al., 2014). Conversely, probiotics lowered lymphocytes and neutrophil values when compared to the control (Owosibo et al., 2013).

## 5 Conclusions

The results of the present study showed that the use of 4 % kefir, yogurt, and probiotic at recommended levels in water had beneficial effects on the growth performance, intestinal bacteria population, and blood biochemical parameters in male and female broiler chickens. Consequently, these experimental results showed that 4 % kefir and yogurt, as kinds of fermented milk products, can be considered synthetic growth-promoter-like commercial probiotics in broiler chickens.
